# A Remedy for the Warping of Plates

**Published:** 1854-01

**Authors:** 


### A REMEDY FOR THE WARPING OF PLATES.

It will be seen by an advertisement in the present number of the Register that Drs. Rickey, of Keokuk, Iowa, and Crider, of Lancaster, Ohio, offer the Profession a remedy for this great difficulty. Perhaps, among the great variety of remedies offered, this is the most certain, for it strikes at the cause itself, and by not soldering at all, gives no chance for warping.

Those having much trouble in the warping of their plates, we have no doubt, will find this an efficient remedy. The plan consists in soldering the teeth to a very narrow gold plate, on which only the lingual half of the heels of the teeth shall rest. This plate extends within, merely sufficient for the basis of the artificial denture and receive some five or six rivets. This is adjusted to the alveolar border on the suction plate, and this having been swedged to fit the mouth, and left in this condition, without annealing, the teeth, with the narrow plate, is rivited on. This riviting is done on the metalic cast. The suction plate may be rimmed as in any other case. Before riviting, the interspaces between the teeth, plate, &c., are filled with a solution of gutta percha in chloroform, and the entire operation made as tight and close as possible. The question may be asked, will this preparation of gutta percha last? For some time, we have no doubt, it will, and while it does, it will be more pleasant than confined particles of food.

It will be seen that Drs. Rickey & Crider have patented this plan of operating. Not having as yet tried the method, we speak only from the assurance of friends and the apparent reasonableness of the plan. We believe the two plates should be swedged together, then made to adhere while the teeth are ground in. The narrow plate and teeth are then put in plaster and sand, and when the teeth are backed and soldered, trimmed and burnished, the job is ready for riviting.

